# Reliability of individual differences in distractor suppression driven by statistical learning

**DOI:** 10.3758/s13428-023-02157-7

**Published:** 2023-07-25

**Authors:** Yavor Ivanov, Jan Theeuwes, Louisa Bogaerts

**Affiliations:** 1 Vrije Universiteit Amsterdam, Van der Boechorststraat 7, 1081 BT Amsterdam, The Netherlands; 2 Ghent University, Ghent, Belgium

**Keywords:** Attentional capture, Distractor suppression, Statistical learning, Individual differences, Test reliability

## Abstract

A series of recent studies has demonstrated that attentional selection is modulated by statistical regularities, even when they concern task-irrelevant stimuli. Irrelevant distractors presented more frequently at one location interfere less with search than distractors presented elsewhere. To account for this finding, it has been proposed that through statistical learning, the frequent distractor location becomes suppressed relative to the other locations. Learned distractor suppression has mainly been studied at the group level, where individual differences are treated as unexplained error variance. Yet these individual differences may provide important mechanistic insights and could be predictive of cognitive and real-life outcomes. In the current study, we ask whether in an additional singleton task, the standard measures of attentional capture and learned suppression are reliable and stable at the level of the individual. In an online study, we assessed both the within- and between-session reliability of individual-level measures of attentional capture and learned suppression. We show that the measures of attentional capture, but not of distractor suppression, are moderately stable within the same session (i.e., split-half reliability). Test–retest reliability over a 2-month period was found to be moderate for attentional capture but weak or absent for suppression. RT-based measures proved to be superior to accuracy measures. While producing very robust findings at the group level, the predictive validity of these RT-based measures is still limited when it comes to individual-level performance. We discuss the implications for future research drawing on inter-individual variation in the attentional biases that result from statistical learning.

## Introduction

One of the many ways in which memory guides our perceptual experiences and actions is evidenced by so-called statistical learning (SL): the ability to pick up and utilize regularities across time and space present in the sensory input. This type of learning is unintentional and largely implicit (Perruchet & Pacton, 2006); it runs "in the background", seeking out the structure of the world around us, thus making it predictable and better manageable. SL mechanisms were first described in the context of language (Saffran et al., 1996; see Erickson & Thiessen, 2015; Romberg & Saffran, 2010 for reviews) but have since been implicated in various other domains of cognition such as motor learning (e.g., Monroy et al., 2019), scene and object perception (e.g., Graham & Redies, 2010; Võ et al., 2019) (for discussion see Bogaerts et al., 2020). Recently, there has been a rapidly growing interest in the role of statistical learning in modulating attentional selection (e.g., Geng & Behrmann, 2002; Jiang et al., 2013; Goschy et al., 2014; Ferrante et al., 2018; Wang & Theeuwes, 2018a, b, 2020; Gao & Theeuwes, 2020; Di Caro et al., 2019; van Moorselaar & Slagter, 2019; Duncan & Theeuwes, 2020; Di Caro & Della Libera, 2021; for reviews see Awh et al., 2012; Theeuwes, 2019; Luck et al., 2021).

### SL modulates visuospatial attention

**Fig. 1:**
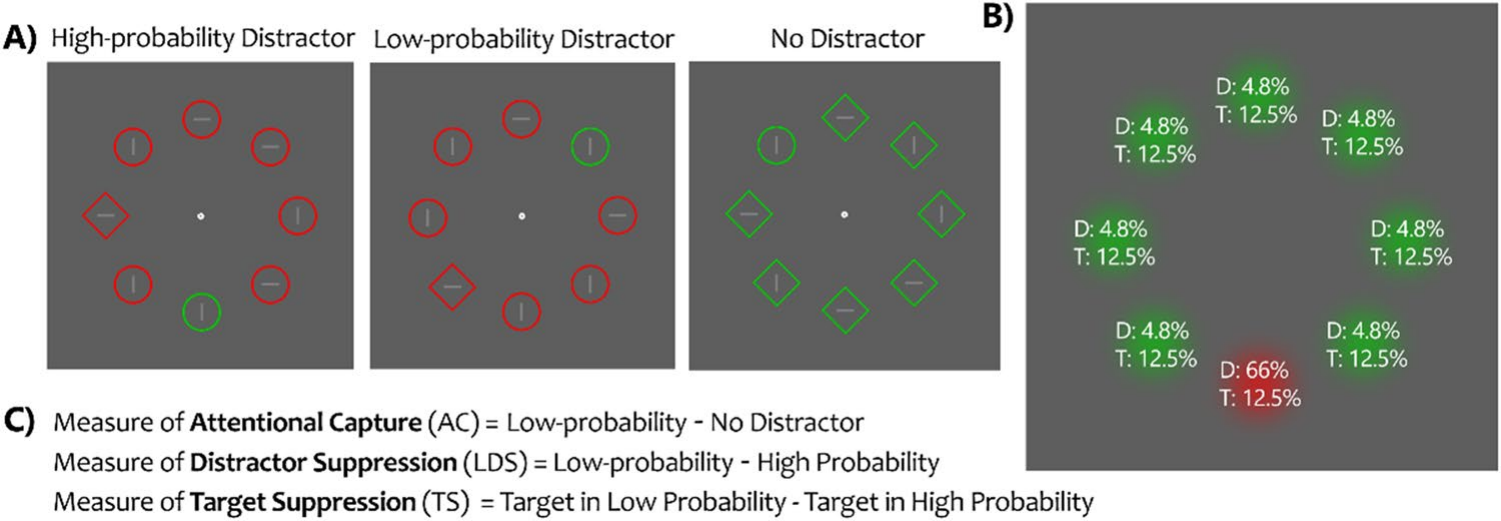
**A** Illustrative examples of the three possible Distractor Conditions used to study learned distractor suppression in the additional singleton paradigm. On every trial, participants look for the uniquely shaped object, and respond based on the orientation of the line within it. **B** Spatial distribution of stimuli in the illustrative display. Percentages show the probability of a singleton distractor (D) or a target (T) appearing at a particular location. **C** Description of how the measures of Attentional Capture (AC); Learned Distractor Suppression (LDS); and Target Suppression (TS) are calculated. A similar computation can be done for accuracy scores instead of RTs

Visual perception must be selective, as we are confronted with large amounts of sensory input. How exactly this selection occurs is an issue of ongoing debate (for a recent review see Luck et al., 2021), yet what multiple recent studies have shown is that statistical regularities lead to modifications of the attentional priority of spatial locations. On the one hand, studies showed that attention is automatically drawn towards locations that have regularly contained task-relevant or rewarding stimuli in the past (e.g., Geng & Behrmann, 2002, 2005; Failing & Theeuwes, 2014, 2018; Ferrante et al., 2018; Jiang, 2018). On the other hand, regularities concerning distracting, task-irrelevant stimuli lead to the suppression of locations that have previously frequently contained them (Ferrante et al., 2017, 2018; Wang & Theeuwes, 2018a, b; van Moorselaar & Slagter, 2019; van Moorselaar & Theeuwes, 2021, etc.). For example, Wang & Theeuwes (2018a) used the additional singleton paradigm (e.g., Theeuwes, 1992), in which participants searched for a uniquely shaped target in a display, with one non-target stimulus occasionally having a unique color (i.e., singleton distractor) (see Fig. 1). Decades of research show that singleton distractors interfere with the search for a target stimulus, as evidenced by slower response times (RTs) when a singleton distractor is present on the display versus when it is not (i.e., stimulus-driven capture; for review see Theeuwes, 2019). Critically, Wang and Theeuwes (2018a) manipulated the probability with which the singleton distractor appeared across the locations on the display, with one location being much more probable than other locations. Their results, together with those of other studies using a similar frequency manipulation (e.g., Ferrante et al., 2018; Zhang et al., 2022; van Moorselaar & Slagter, 2019; Di Caro & Della Libera, 2021) showed that the interference caused by the singleton distractor was significantly reduced when the singleton appeared in its high-probability location, versus in any of the other locations. It was concluded that through SL, the high-probability distractor location became suppressed, and this occurred within the span of several trials and without much, if any, conscious awareness (e.g., Wang & Theeuwes, 2020; Gao & Theeuwes, 2022). Moreover, this learning can occur in the absence of explicit top-down attention (Duncan & Theeuwes, 2020), and the suppression is proactive in nature suggesting that it occurs before display onset (Wang et al., 2019; Huang et al., 2021; van Moorselaar & Slagter, 2019).

In summary, there is ample evidence for an implicit mechanism for *Learned Distractor Suppression* (from now on abbreviated as *LDS*). However, with few exceptions (e.g., Ferrante et al., 2018; van Moorselaar & Slagter, 2019) this line of work exclusively focused on group-level effects, treating individual differences as unexplained variance. Yet, this variance might be of great interest as recent reports revealed the existence of stable individual differences in SL, with predictive validity. In what follows we first discuss the findings on SL as an individual ability, thereafter we consider individual differences more specifically in the context of learned biases in attentional selection.

### Individual differences in SL

Whereas the implicit learning of regularities was initially thought of as characterized by low between-subject variability, reflecting mainly measurement noise, more recent findings demonstrated the existence of substantial and meaningful individual differences in SL abilities (see Siegelman et al., 2017, for a recent review). Much of the work in this area was motivated by the hypothesis that individual capacities to incidentally learn regularities in sensory input may be predictive of real-life outcomes such as language skills. In line with this hypothesis, several studies demonstrated that one’s ability to pick up on transitional probabilities in streams of visual elements (typically referred to as Visual Statistical Learning, or VSL; e.g., Turk-Browne et al., 2005) correlates with language outcomes such as reading ability (e.g., Arciuli & Simpson, 2012a, b), language processing (Misyak et al., 2010), and second language acquisition (e.g., Frost et al., 2013) (see Siegelman, 2020, for a recent review), but also with non-linguistic outcomes such as feature-comparison skills (Growns & Martire, 2020) or even social competency (Parks et al., 2020). More generally, the study of individual differences provided critical insights regarding the mechanisms underlying SL. For example, SL abilities were found to have a near-zero correlation with general measures of intelligence or working memory (Siegelman & Frost, 2015; Kaufman et al., 2010), as such they may explain variance in cognitive tasks above and beyond intelligence. Correlational approaches have further helped to clarify the nature of the learning mechanisms by providing evidence for domain-specificity, showing high correlations between performance on SL tasks within a same sensory modality but low correlations on SL tasks in different modalities (e.g., Redington & Chater, 1996; Tunney & Altmann, 1999; Gomez et al., 2000; for a review see Frost et al., 2015; Siegelman, 2020). Finally, Growns et al. (2020) explored the componentiality of SL mechanisms by studying individual differences in SL tasks that differed in the nature of the regularities with which participants were presented: regularities were either distributional (x is more frequent than y) or conditional (after x follows y), and either spatial, or nonspatial. They observed substantial shared variance across all tasks, as well as a portion of variance unique to each task. This correlational evidence was taken to suggest that VSL performance is the result of the interplay between a unified mechanism for extracting different types of visual regularities, and an individual’s ability to extract a specific type of regularity.

### Individual differences in SL modulating visuospatial attention

Surprisingly, despite the large interest in individual differences in SL in recent years, inter-individual variation remains largely unexplored when it comes to the modulation of visuospatial attention by statistical regularities. In the current study, we investigated whether different individuals reliably show different levels of learned distractor suppression within a given visual environment that is characterized by a distributional regularity. This may be expected as individuals are not all equally sensitive to such a regularity. If they do, these individual differences may elucidate the factors driving LDS. For example, LDS has been observed using a classic parallel-search task as discussed above, but a similar bias has also been observed in serial-search (e.g., Wang & Theeuwes, 2018b). Correlating performance between a similar parallel and a serial visual search paradigm with the same distractor regularity will allow us to examine whether LDS in the two types of searches rely on a single shared mechanism. Moreover, individual differences in attentional capture and LDS may be predictive of other cognitive capacities and real-life outcomes that depend on the optimized deployment of visual attention (e.g., visual perception, navigation, driving, reading, etc.). A better understanding of the individual differences of the LDS effect will not only allow for a better understanding of the attentional control system but may also aid in the development of diagnostic tools or interventions for various attention-related disorders.

The LDS effect is, operationally, defined as a reduction in the *attentional capture* (from now on abbreviated as *AC*) of distractors appearing at the frequent location. It is worth noting that several studies have investigated the individual reliability of measures of AC driven by bottom-up (e.g., salience) and top-down (e.g., inter-trial priming) factors (e.g., Weichselbaum et al., 2018), or reward (Anderson & Kim, 2019b). For example, Weichselbaum and colleagues (2018) showed that the commonly used RT measure of AC (i.e., aggregate difference scores of RTs between distractor absent vs. present conditions) and eye-tracking measures of AC (i.e., target fixation latency) were stable over a one- or four-week period with high test–retest correlation coefficients of around .60 to .90 (see also Anderson & Kim, 2019a). However, the reliability of capture was investigated in the absence of statistical regularities regarding distractors.

To the best of our knowledge, no previous studies have tested whether LDS can be measured reliably within individuals. As argued above, there are clear incentives for exploring individual differences in LDS. Several recent studies have indeed tried correlating individual LDS scores with other metrics. Ferrante et al. (2018), for example, correlated individual distractor suppression scores with target selection scores and found an anti-correlation, which was interpreted as support for a unitary mechanism of spatial prioritization, whereby ignoring and selecting are two sides of the same coin. Moorselaar et al. (2021) asked whether individuals show less spatial distractor learning if their distractor rejection is efficient in the absence of spatial regularities and showed that, indeed, measures of attentional capture positively correlated with LDS measures within individuals. In recent theoretical work, it was explicitly suggested that one way to tackle the question of whether statistical learning as observed in LDS is driven by the same learning mechanism as the one tapped in other visual statistical learning tasks “[…] would be to investigate individual differences: systematic positive correlations between performance across a range of VSL tasks such as embedded triplet learning and learned distractor suppression would be consistent with the view of VSL as a unitary learning system” (Theeuwes et al., 2022, p. 862). However, the success of an individual differences approach is entirely dependent on the use of tasks and measures that are suited to study individual performance. Hence it is critical to first establish whether the measures used to index it are reliable at an individual level (Hedge et al., 2018). The fact that they produce robust effects on the group level should not be taken as evidence that they measure individual performance reliably, as psychometric considerations depend on the level of measurement. There might even be a paradox here as “[…] the very reason such [classic] tasks produce robust and easily replicable experimental effects—low between-participant variability—makes their use as correlational tools problematic.” (Hedge et al., 2018, p. 1166). Another complication to using group-level paradigms to study individual differences is the use of difference scores obtained by taking a performance measure in an experimental condition and subtracting from it a performance measure in a baseline condition. Difference scores, although widely applied in the study of attention and cognition more generally, tend to suffer from low reliability (Draheim et al., 2019).

As pointed out in Draheim et al. (2019), in cognitive tasks where component scores are highly correlated with each other (e.g., congruent and incongruent trials in the Stroop task; high probability and low probability trials in the statistical learning singleton paradigm), the difference scores they produce typically display much lower reliability than the components themselves. However, most research questions in attention research are difficult to answer just by looking at RT-based component scores. To exemplify, in the case of learned distractor suppression, we cannot estimate if someone is a better or worse learner based only on their RTs for high probability trials as an individual might simply be faster at search in general or might be less impacted by the presence of a distractor independent of its location. This is why RT-based difference scores are still widely used for measuring individual-level cognitive capacities in both research and clinical settings (Draheim et al., 2019). Various ways to address the psychometric issues with difference scores have been proposed, such as binning or using integrated RT/accuracy measures (e.g., Hughes et al., 2014; Vandierendonck, 2017; Liesefeld & Janczyk, 2019; etc.). However, many recent studies exploring various distractor suppression effects at an individual level have relied on correlations between difference scores (e.g., Noonan et al., 2016; Ferrante et al., 2018; van Moorselaar & Slagter, 2019; Heuer & Schubö, 2020; Zhang et al., 2022; etc.), hence our focus will be on the assessment of their reliability.

Note that the reliability of a measure is critical to any correlational approach as the true correlation between any two measures is upper-bounded by their respective reliability. Thus, correlational studies exploring the relationship between LDS and other cognitive measures will most likely result in null or spurious findings if one or more of the measures are not sufficiently reliable.

### Current study

The purpose of the present study was to establish whether LDS, measured via difference scores in manual RTs and accuracy, is a reliable and stable individual-level measure. As a proxy to LDS, we also tried to assess the reliability of the target suppression effect in conditions in which the search target appeared in the high-probability distractor location. As secondary goals, we wanted to assess the reliability of the AC measure and compare that to estimates in previous studies. To achieve this, we used a typical test–retest method, where we invited the same sample of participants to complete the task designed by Wang and Theeuwes (2018a) in two sessions separated by a 2-month period. The study was conducted through the Internet. We first assess the effects on a group level, where we expect to replicate the classic finding that distractors, when present, capture attention and the more recent finding that this capture is reduced when singleton distractors appear at a high-probability location versus any other location. Turning to the individual level, we first assess the split-half reliability of each measure (i.e., their internal consistency within a test session). Crucially, by having participants do the task twice, we can also correlate the individual measures obtained in each session with each other. If we find high test–retest reliability (i.e., a high correlation between the measures in Sessions 1 and 2), it will provide evidence that AC and DS, as currently measured, are stable over time. Since reliability is a necessary condition for predictive validity, establishing reliability would open the possibility to investigate the factors underlying individual differences in these measures in the future. If only weak or no correlations are found between both sessions, this may point to either a lack of stability (i.e., it is not a stable individual characteristic) or to the improper measurement of the phenomena on the individual level.

## Method

The study was approved by the Ethical Review Committee of the Faculty of Behavioral and Movement Sciences of Vrije Universiteit Amsterdam.

### Participants

Participant recruitment was done through Prolific (www.prolific.co; Palan & Schitter, 2018). The minimal sample size was planned based on previous studies of SL task evaluation and development (e.g., Siegelman et al., 2017; Bogaerts et al., 2018) and published statistical guidelines for the design of reliability studies (e.g., Brysbaert, 2019; Shoukri et al., 2004; Schönbrodt & Perugini, 2013), while taking into consideration the generally large size of the learning effect we are measuring (*d* is typically around 0.8 in most studies). We invited more participants than planned as we expected that a large proportion of the online data will have to be excluded. One hundred and thirty-six participants signed up and completed Session 1. Data from 18 participants were removed due to performance below chance level or abnormally long mean response times (i.e., >2000 ms). Therefore, all analyses of Session 1 data were performed with a sample of 118 participants (57 female, mean age: 30). The participants that remained after data exclusion were invited for Session 2. Seventy-nine of the original participants signed in and completed Session 2. Data cleaning procedures were repeated for this subset of participants, excluding one participant with an incomplete dataset and one with poor performance. Thus, analyses of Session 2 data and all test–retest correlations are based on a sample of 77 participants (33 female; mean age = 30.2).

### Apparatus and stimuli

The experiment was designed using OpenSesame (Mathôt et al,, 2012) with the OSWeb extension for online experiments, and hosted online using Jatos (Lange et al., 2015). Because the experiment took place online, participants used their own desktop or laptop computers to do the task and some factors of the setup (e.g., screen size, lighting, and seating conditions) could not be controlled. Item sizes and colors are hence reported in pixels and RGB values (red/green/blue).

As illustrated in Fig. 1, the visual search array consisted of eight stimuli with different shapes: either a circle with a radius of 90 px among diamonds subtending 160 x 160 px, or vice versa. These were displayed on a dark grey background (RGB: 94, 94, 94). Each shape had either a red (255, 0, 0) or green color (0, 200, 0). Stimuli were centered around a fixation dot with a radius of 8 px. Each stimulus contained a 12 x 82 px grey line within, oriented either horizontally or vertically.

### Procedure and design

Each trial began with the presentation of a fixation dot for a random duration between 500 and 750 ms. Then the search display was shown for 3000 ms or until response. Participants had to search for the one uniquely shaped target (e.g., a circle among diamonds, or vice versa), and indicate the orientation of its inner grey line by pressing either the UP or DOWN arrow keys for a vertical or horizontal response, respectively.

The target was present on each trial, and its shape was randomly determined. Half of the trials had a diamond target, and the other half had a circle target. A uniquely colored distractor singleton was shown on 66% of the trials. In half of the trials, the distractor was red and surrounded by green stimuli, and in the other half, it was green and surrounded by red stimuli. As in Wang and Theeuwes (2018a, b), one location had a high probability of containing the singleton distractor (i.e., 66% of singleton distractor present trials; 44% of all trials); all other locations had a low probability (4.8% in each location) of containing the singleton distractor. The high-probability location was randomly assigned for each participant. In trials in which a singleton distractor was absent, the location of the target was randomly determined.

At the start of the test session, examples of displays were shown, and participants were instructed to search for a unique shape. There was no mention of the regularity, and there was no emphasis made on speed or accuracy. After the instructions, participants completed 24 practice trials in which the distractor singleton was already more likely to appear at the high-probability location. If their accuracy was below 55% after practice, they were asked to repeat the practice block again. The experiment itself had three blocks each consisting of 144 trials. Between blocks participants could take a self-paced break.

The same participants were invited to participate again two months and three days after they completed the first session. All participants completed the second session within the same day they were invited, with the exception of four participants who started and completed it on the next day. The task in Session 2 was identical to Session 1. Notably, as the selection of the high-probability location was random for each participant and in each session, the two sessions typically had a different high-probability location, however, for some participants, the same location was repeated (13% of participants).

## Results

Data were processed (e.g., applying exclusion criteria) using a custom Python script and statistical analyses were performed using Python, JASP (JASP team, 2022) and R (publicly available at https://osf.io/stvpf/?view_only=31c7881b3f534686ac3589504812f057).

Analyses of Session 1 have a different number of participants than analyses of Session 2 due to the high dropout rate (*n* = 118 and *n* = 77, respectively). For RT analyses, only trials with a correct response were included (92% of all trials in Session 1; 94% of all trials in Session 2). Furthermore, trials on which RTs were 2.5 SD below or above the mean (within-participant) were removed.

### Group-level analyses

#### Response times

**Fig. 2:**
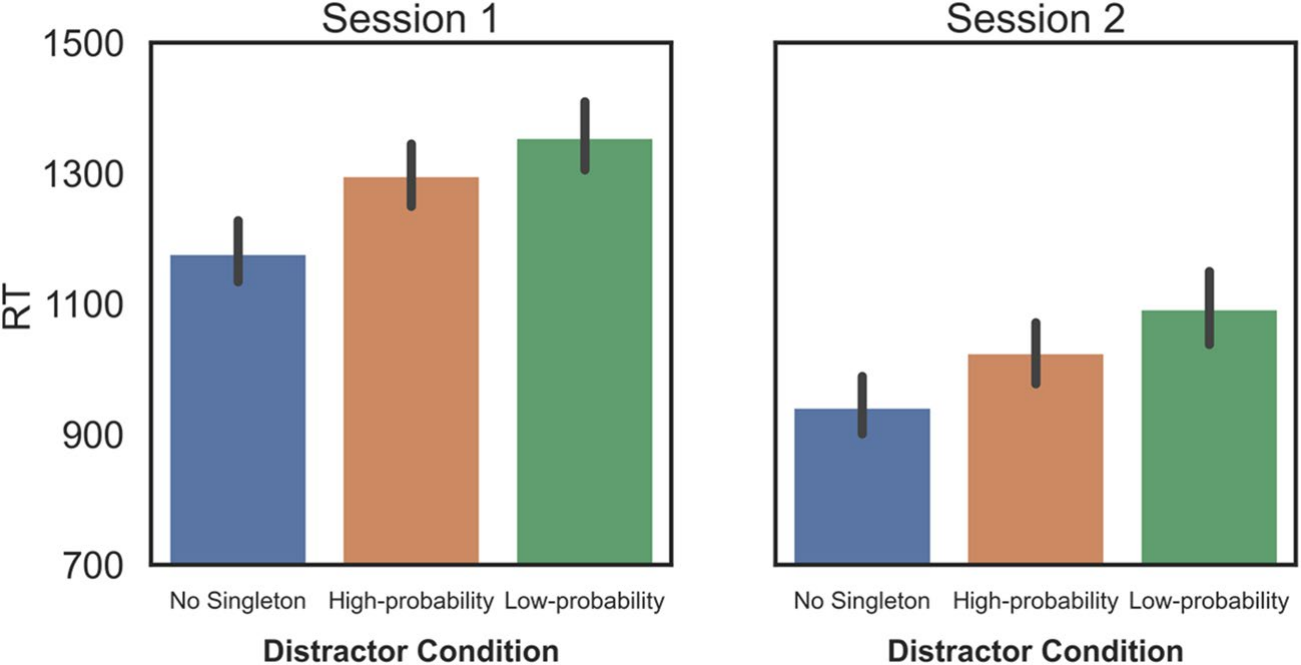
Group-level RT results for the three distractor conditions. Error bars represent within-subject 95% confidence intervals

Group-level differences in average RTs (see Fig. 2) were assessed using a repeated-measures ANOVA with a factor for Distractor Condition (High-probability vs. Low-probability vs. No Distractor). When applicable, the degrees of freedom of the Greenhouse–Geisser sphericity correction are reported instead of the original degrees of freedom (*n* = 118 in Session 1 and *n* = 77 in Session 2). There was a significant main effect of Distractor Condition in both Session 1 (*F(1.933, 226.17)* = 184.36, *p* < 0.001, *η*^*2*^_*p*_ = 0.61) and in Session 2 (*F(2,152)* = 202.15, *p* < 0.001, *η*^*2*^_*p*_ = 0.727). Post hoc comparisons in Session 1 show that responses in the No Distractor condition were 119 ms (8.4 SE) faster than in the High-probability condition (*t(117)* = 14.21, *p <* 0.001, *d* = 1.31), and 171 ms (9.88 SE) faster than in the Low-probability condition (*t(117)* = 17.38, *p <* 0.001, *d* = 1.6). Post-hoc comparisons in Session 2 show that responses in the No Distractor condition were 81.7 ms (6.4 SE) faster than in the High-probability condition (*t(76)* = 12.81, *p < *0.001, *d* = 1.46), and 147 ms (8.4 SE) faster than in the Low-probability condition (*t(76)* = 17.49, *p < *0.001, *d* = 1.99). Most importantly, in Session 1 RTs in the High-probability condition were 52 ms (9.17 SE) faster than in the Low-probability condition (*t(117)* = 5.72, *p < *0.001, *d* = 0.526); whereas in Session 2 this difference was 65 ms (7.09 SD) and also significant (*t(76)* = 9.27, *p < *0.001, *d* = 1.057). In general, the results show that the singleton distractors interfered with search, and that interference by distractors at the high-probability location was indeed suppressed relative to other locations in both experimental sessions.

#### Accuracy

**Fig. 3:**
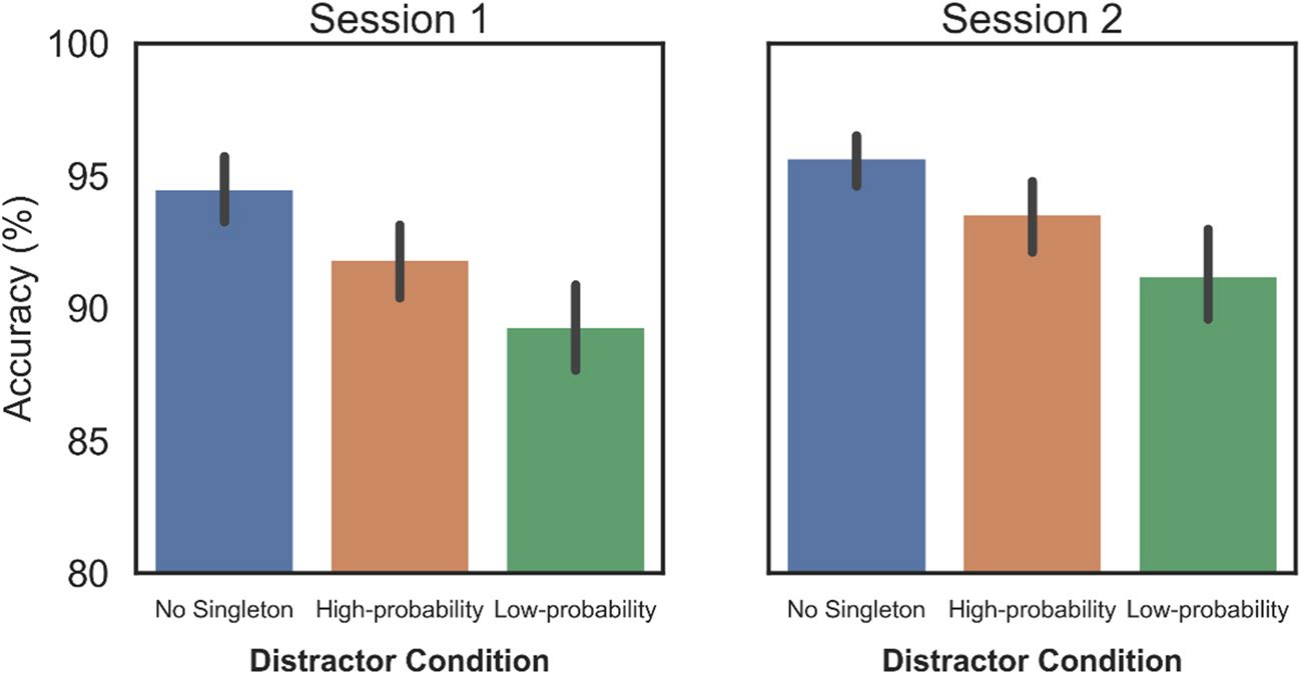
Group-level accuracy results for the three distractor conditions. *Error bars* represent within-subject 95% confidence intervals

Group-level differences in average Accuracy (see Fig. 3) were assessed using a repeated-measures ANOVA with a factor for Distractor Condition (High-probability vs. Low-probability vs. No Distractor). The analyses suggest that accuracy significantly differed between the three possible Distractor Conditions in Session 1 (*F(2,234)* = 53.68, *p* < 0.001, *η*^*2*^_*p*_ = 0.32) and also in Session 2 (*F(2,152)* = 38.68, *p* < 0.001, *η*^*2*^_*p*_ = 0.34). Post hoc tests reveal that in Session 1 accuracy in the No Distractor condition was 2.7% higher than in the High-probability condition (*t(117)* = 7.02, *p < *0.001, *d* = 0.647), and 5.2% higher than in the Low-probability condition (*t(117)* = 8.89, *p < *0.001, *d* = 0.819). In Session 2, post hoc tests reveal that accuracy in the No Distractor condition was 2.1% higher than in the High-probability condition (*t(76)* = 4.57, *p < *0.001, *d* = 0.521), and 4.4% higher than in the Low-probability condition (*t(76)* = 7.94, *p < *0.001, *d* = 0.906). Crucially, accuracy in the High-probability condition was 2.5% higher than in the Low-probability condition (*t(117)* = 4.86, *p < *0.001, *d* = 0.448) in Session 1; whereas in Session 2 this difference was 2.3% and also significant (*t(76)* = – 4.76, *p < *0.001, *d* = 0.543). Complementing the RT results, we see that participants gave more accurate responses when distractors appeared in the high-probability location versus other locations.

#### Target suppression

**Fig. 4:**
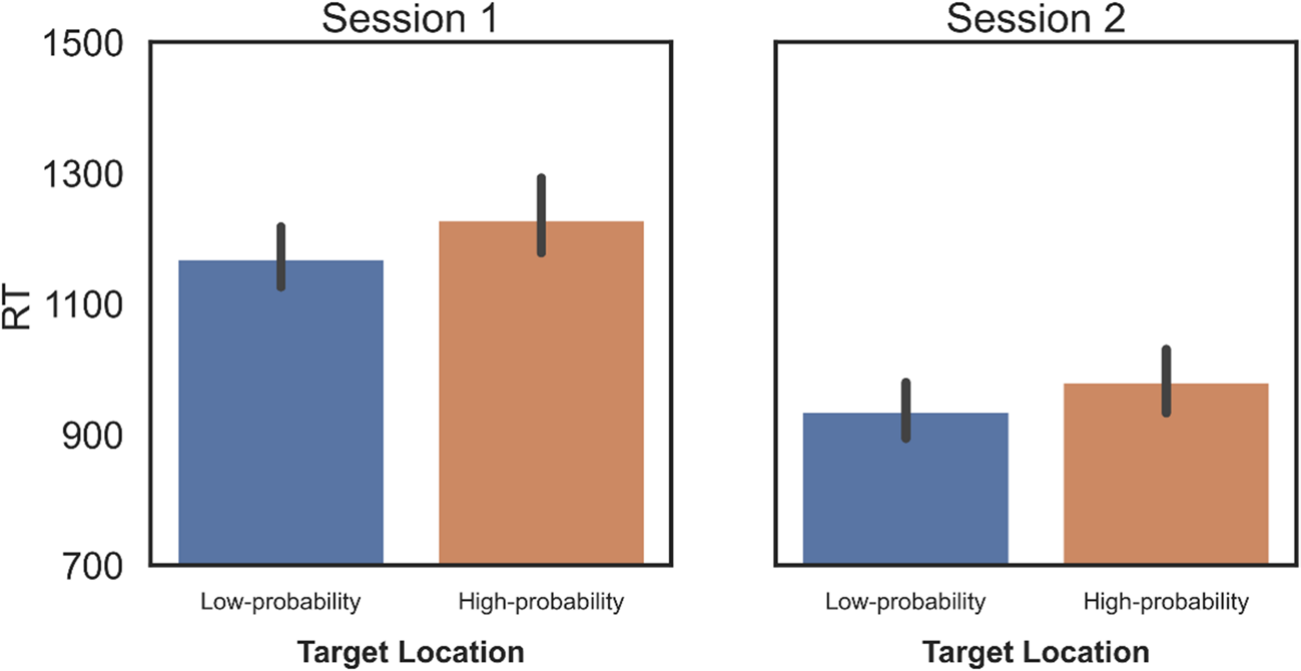
Group-level RT results for trials in which the target appeared in the high-probability distractor location versus trials in which it did not. *Error bars* represent within-subject 95% confidence intervals

Additional analyses were done to see if targets appearing at the high-probability distractor location are also suppressed, as was found in the original Wang and Theeuwes (2018a, b) paper (see Fig. 4). On the subset of trials in which the singleton distractor was not present we performed a paired-samples *t* test to test the difference between RTs on trials in which targets appeared at the high-probability distractor location vs. at any other location. In Session 1, targets in the high-probability location were responded to 65 ms (14.87 SE) slower than targets appearing elsewhere (*t(117)* = 4.38, *p < *0.001, *d* = 0.404); in Session 2 this difference was 49 ms (10.67 SE) and was also significant (*t(76)* = 4.66, *p < *0.001, *d* = 0.532).

When using Accuracy as a dependent variable, paired-samples *t* tests showed no evidence of target suppression in either session (Session 1: *t(117)* = – 0.849, *p = *.398, *d* = – 0.078, Session 2: *t(76)* = – 0.395, *p = *.698, *d* = – 0.045).

In summary, we successfully replicated the original pattern of results showing suppression of attentional selection of distractors and targets when they appear at the High-probability distractor location versus any Low-probability locations (e.g., Wang & Theeuwes, 2018a).

### Individual-level analyses

#### Between-subject variance in AC, LDS, and target suppression measures

**Fig. 5:**
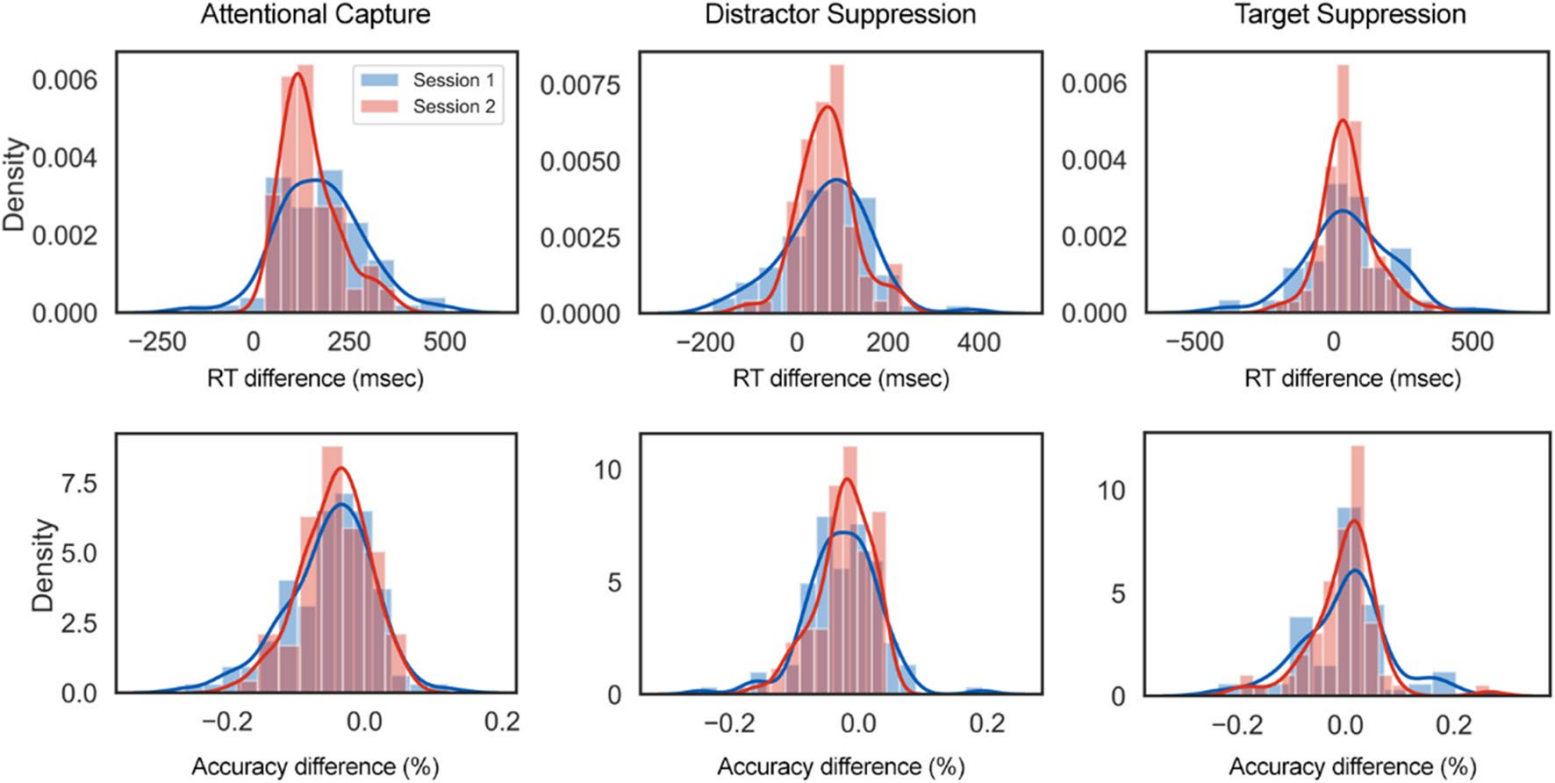
Probability Density Functions for the AC (*left*), LDS (*middle*), and Target Suppression (*right*) measures, split between the two test sessions. The *blue color* represents Session 1 and the *red color* represents Session 2. The *top row* depicts RT measures, and the *bottom row* depicts Accuracy measures. The *smooth curves* represent kernel density estimates

For our measures of AC, LDS, and Target Suppression we computed the average response time or accuracy difference score between the relevant conditions, separately for each participant (see Fig. 1). Thus, AC was calculated as the average difference between the Low-probability and No Distractor conditions. LDS was calculated as the average difference between the High-probability and Low-probability conditions. Target suppression was calculated as the average difference in no-distractor trials in which the target was presented in the high-probability location versus trials in which it was presented elsewhere. Figure 5 shows the distribution of these measures for each of the two sessions, where substantial inter-individual variability was observed.

#### Split-half reliability

As a first step towards assessing the reliability of our AC, LDS, and target suppression measures, we document the split-half reliability to evaluate their internal consistency (see Table 1 for a summary of the results). The ‘splithalf’ function from the eponymous R library (Parsons, 2021) was used to compute split-half reliability estimates via a bootstrapping permutation approach, by randomly splitting the data into two halves (with 5000 permutations) and finding the mean difference. As discussed by Pronk et al. (2022), the bootstrap permutation approach is possibly better suited for estimating reliability coefficients of RT difference scores than standard odd-even trial splits. We report the Spearman–Brown corrected estimates derived from the bootstrapped distributions and 95% confidence intervals (CIs). Bivariate outlier detection was performed using the outliers_mcd function found in the Routliers R library using the Mahalanobis-distance method for multivariate outliers (Leys et al., 2018). Outliers were identified using data from an even-odd trial split per type of measure and session. On average, ten participants were removed per type of measure in each session (range, 5–13).*Values in bold* show the Spearman–Brown corrected *r* coefficient; *values in square brackets* show the 95% CI of the bootstrapped distribution of estimates. *Coefficients in brackets* show the results after bivariate outlier removal.

**Table 1:** Results of the split-half analyses

	Split-half reliability			
	Session 1		Session 2	
Measure	RTs	Acc	RTs	Acc
Attentional Capture	**0.51 (0.25)** [0.35, 0.64]	**0.34 (0.12)** [0.09, 0.54]	**0.67 (0.43)** [0.54, 0.78]	**0.43 (0.28)** [0.20, 0.62]
Distractor Suppression	**0.40 (0.12)** [0.21, 0.56]	0.08 (**–** 0.30) [– 0.21, 0.34]	**0.51 (0.16)** [0.32, 0.67]	0.17 (**–** 0.29) [– 0.15, 0.45]
Target Suppression	**0.39 (0.03)** [0.19, 0.55]	0.23 (0.14) [– 0.05, 0.46]	**0.46 (0.26)** [0.22, 0.65]	0.26 (**–** 0.30) [– 0.03, 0.50]

*First values* show the Spearman–Brown corrected *r* coefficient; *values in square brackets* show the 95% CI of the bootstrapped distribution of estimates. *Coefficients in brackets* show the results after bivariate outlier removal. *Coefficients in bold* are statistically significant at the 95% confidence level

For target-suppression analyses, an additional filtering was applied to the data to ensure that splitting in halves could be performed: individual sessions were removed where the target occurred at the high-probability distractor location less than ten times due to chance. Target-suppression analyses in Session 1 therefore have *n* = 107 instead of *n* = 118.

Results obtained when using all data are summarized in Table 1. When considering all individuals, the split-half method suggests that in both sessions the RT measures of AC, LDS, as well as target suppression show moderate levels of split-half reliability. For comparison, internal consistency scores of standard cognitive tests are typically about .70 or more (Nunnally & Bernstein, 1994). Accuracy scores showed weaker internal consistency than RTs, for AC measures, and were even non-significant for LDS and target suppression.

The removal of bivariate outliers, however, resulted in a drop in reliability coefficients across the board, with only the RT-based measure of AC in Session 2 still having a significant positive correlation (*r* = 0.43, 95% CI: [0.17, 0.63]).

#### Correlations between different measures

Within each session, the RT measures of AC and LDS were correlated with each other (Session 1: *r* = 0.56, *p* < 0.001, 95% CI*:* [0.38, 0.69]; Session 2: *r* = 0.67, *p* < 0.001, 95% CI: [0.53, 0.78]); and so were the accuracy measures (Session 1: *r* = 0.71, *p* < 0.001, 95% CI: [0.58, 0.80]; Session 2: *r* = 0.61, *p* < 0.001, 95% CI: [0.48, 0.73]). By contrast, RT measures of target suppression in a given session did not correlate significantly with their analog measures for distractor suppression (Session 1: *r* = 0.12, *p* = 0.289, 95% CI: [– 0.10 ,0.33]; Session 2: *r* = 0.22, *p* = 0.052, 95% CI: [– 0.002, 0.424]), or with AC (Session 1: *r* = 0.14, *p* = 0.232, 95% CI: [– 0.08 ,0.35]; Session 2: *r* = 0.15, *p* = 0.195, 95% CI*:* [– 0.07, 0.361]).

#### Test–retest reliability

**Fig. 6:**
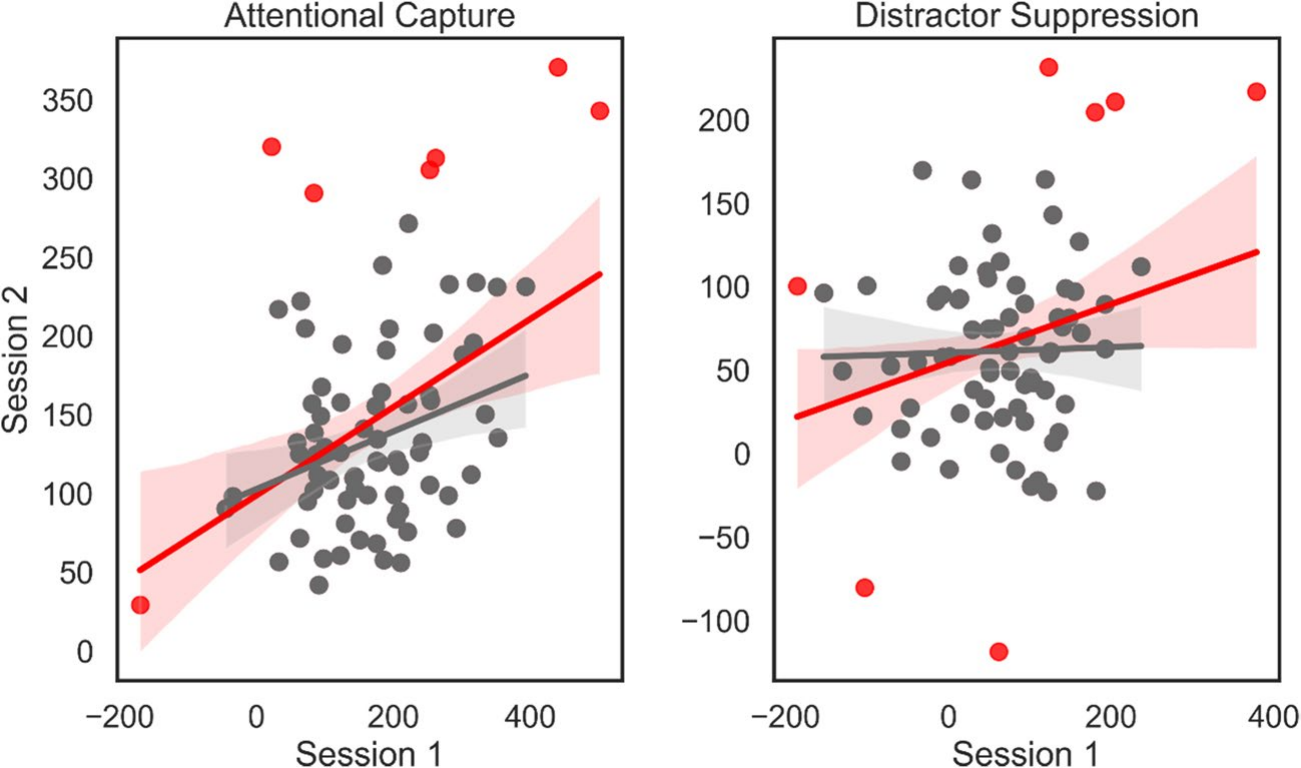
Individual RT difference scores for AC (*left*) and LDS (*right*) between Session 1 (*x*-axis) and Session 2 (*y*-axis). *Red dots* and *red regression lines* represent the detected bivariate outliers and the linear relationship before removing them. The *grey dots* and *grey regression lines* show the data points and linear relationship with bivariate outliers removed. The *shaded area around the linear regression lines* represents bootstrapped 95% CI

Finally, to assess the stability of the AC and LDS measures over a 2-month period, we examined their test–retest reliability. Note that the test–retest analyses are conducted with *n* = 77 due to the dropout between Session 1 and 2. We computed a Pearson* r* coefficient between the Session 1 and Session 2 difference score measures of each individual^1^. The results of the test–retest correlations are presented in Table 2 and Fig. 6. When considering all individuals, RT indices of AC and LDS showed weak to moderate test–retest reliability.

**Table 2:** Results of the test–retest reliability analyses

	Test–retest reliability	
	Across sessions	
Measure	RTs	Acc
Attentional Capture	**0.42** *** (0.31 ***) [0.22, 0.59]	**0.18** [*–* 0.04, 0.39]
Distractor Suppression	**0.26** ** **(0.02)** [0.04, 0.46]	*–* **0.13** [*–* 0.35, 0.09]
Target Suppression	*–* **0.16** [*–* 0.37, 0.06]	*–* **0.085** [*–* 0.30, 0.14]

*Values in bold* show the Pearson *r* coefficients. *Three stars* denote *p* < 0.001, *two stars p* < 0.01. *Values in square brackets* show the 95% CI. *Coefficients in brackets* show the results after bivariate outlier removal

Reliability coefficients were also estimated after removing bivariate outliers using the Mahalanobis-distance method (Leys et al., 2018). In both AC and LDS seven outliers were removed (see Fig. 6). For the RT measure of AC, this resulted in a slight decrease in its test–retest correlation compared to before outlier removal (*r* = 0.31, *p* = 0.007, 95% CI: [0.08, 0.51]) whereas the correlation between RT measures of LDS was now flat (*r* = 0.02, *p* = 0.811, 95% CI: [– 0.21, 0.26]).

#### Mediation analysis

Given the high correlation between AC and LDS measures, and the fact that the former had overall better reliability, it could be that statistical learning had no unique relationship between the two sessions, but what we observed was rather that the statistical learning measures act as a proxy for AC. In other words, it could be that the correlation of statistical learning between the two sessions was mediated by the amount of general distractor interference experienced by an individual. To test this, we conducted a mediation analysis (*n* = 77) in JASP (JASP team, 2022) with the bootstrapped CIs method (1000 iterations). The dependent variable was LDS in Session 2, the independent variable was LDS in Session 1, and Average AC (i.e., the within-participant average AC across the two sessions) was used as a mediator. The direct effect of LDS was not significant (*p = *0.786; 95% CI: [– 0.002, 0.002]) whereas the indirect effect was significant (*p* < 0.001; 95% CI: [0.002, 0.005]).

Overall, test–retest correlation coefficients are much lower compared to the split-half reliability, indicating that participants’ performance on a same measure lacks stability between the initial administration of the task and the retest. Bivariate outlier removal resulted in test–retest estimates dropping to levels that are well below acceptable (Nunnally & Bernstein, 1994), especially so for the learning measures. A mediation analysis further suggested that the relationship between Session 1 and Session 2 distractor suppression measures is better explained indirectly by taking into account the individuals’ average distractor interference.

## Discussion

The statistical learning (SL) literature suggests that humans are able to implicitly learn various types of visual regularities, and that their ability in doing so may be related to real-life outcomes in the domain of language and beyond. A multitude of recent studies show that SL mechanisms are also involved in modulating attentional selection: attention can be suppressed at locations that have previously been associated with a high probability of containing distracting stimuli. Attentional capture and its modulation by the presence of spatial statistical regularities has mostly been examined at the group level, but there is an increasing interest in individual differences.

### Reliability: a necessary condition for individual-level research

Several recent studies used a correlational approach, for example, to provide evidence for a shared mechanism underlying distractor suppression and target suppression (Ferrante et al., 2018). Indeed, we have argued that the study of individual differences is a promising avenue to help elucidate the factors driving LDS and its relation to other forms of statistical learning (Theeuwes et al., 2022). The consideration of attentional capture and LDS at the individual level is further enticing as they may be predictive of other cognitive capacities and real-life outcomes that depend on the optimized deployment of visual attention. However, the success of such a research agenda depends on the use of reliable measures of individual performance. This seems straightforward, yet the implicit assumption of reliability at the individual level for measures that produce robust effects on the group level has plagued the attention literature (Anderson & Kim, 2019b) as well as the cognitive sciences more broadly (Hedge et al., 2018). More generally, documenting reliability can help researchers decrease the impact of measurement error, increase reproducibility, and make informed methodological choices (Farkas et al., 2023). We therefore set out to evaluate the reliability of the typical measures of statistically learned spatial suppression as used in a series of recent studies (e.g., Wang & Theeuwes, 2018a, b; Wang & Theeuwes, 2020; Gao & Theeuwes, 2020; Di Caro et al., 2019; van Moorselaar & Slagter, 2019; Duncan & Theeuwes, 2020; Huang et al., 2021; Xu et al., 2021; de Waard et al., 2022; Bogaerts et al., 2022; Li et al., 2022; etc.).

In the present study, we successfully replicated the original group-level findings (Wang & Theeuwes, 2018a), showing that responses were faster and more accurate when distractors appeared at high-probability locations versus low-probability locations. More importantly, our results highlight the existence of substantial individual differences in both attentional capture (AC) and LDS. Using a prototypical version of the additional singleton task in which target and distractor features (i.e., red or green circles or diamonds) swap randomly, we found that the commonly used RT and accuracy measures of AC show moderate internal consistency. Additionally, distractor interference as seen in response times, but not accuracy, was somewhat temporally stable over the course of two months. This is consistent with previous investigations (e.g., Weichselbaum et al., 2018) which showed that AC can be reliably measured using both button presses and eye-tracking (with eye-tracking providing more reliable estimates due to its increased sensitivity). Our study extends these findings by showing that AC is at least to some extent temporally stable even when measured in an online study, and at a longer time period (2 months as opposed to the 1-week and 1-month periods used in Weichselbaum et al., 2018). The fact that we find reliability coefficients smaller than the ones reported in Weichselbaum et al. (which were in the range of .60 to .90), most likely results from differences in the experimental settings (online vs. lab) and possibly the delay between test and retest (2 months vs. 1 month). Because the current study was conducted online, participants used their own computer. If individuals used the same technical setup in both sessions, between-subject differences in setup (e.g., monitor size, color quality) could, in theory, affect the size of an individual’s capture effect and contribute to an overestimation of test–retest reliability estimates. Yet, the fact that we observed robust capture and found a lower reliability estimate for AC compared to lab-based studies suggests that it did not have a substantial influence.

The main focus of the study, however, was the reliability of the measure of statistical learning regarding the distractor location (LDS) and the resulting suppression of targets appearing at that location (target suppression). Split-half analyses revealed that the RT measures of LDS were moderately stable within a session. In general, we found that response time measures were more reliable than accuracy measures, likely because accuracy was at ceiling levels, greatly reducing inter-individual variance. After bivariate outlier removal, split-half correlations dropped substantially, with only the RT-based metric of attentional capture in Session 2 still having a significant positive correlation. All in all, this suggests that the internal consistency of both RT and Accuracy measures of attentional capture and distractor suppression is weak or even absent. In line with the split-half analyses, the test–retest correlations suggest weak to near-zero reliability for measures of distractor and target suppression (whether based on RT or accuracy), and modest reliability across sessions only for the measure of attentional capture. From a theoretical perspective, this suggests that whereas the ability to filter out color singleton distractors as measured in the current singleton paradigm may be somewhat stable across sessions, the modulation of attentional capture by learning a regularity about the likely spatial location of a color singleton is not. One possible interpretation of this result is that the ability to suppress distractors by exploiting a learnable regularity regarding the likely location of distractors is highly time- and context-sensitive, as has been argued for other forms of learning (Farkas et al., 2023). However, low test–retest correlations do not necessarily imply that the construct that is measured is not a stable characteristic of an individual: the present findings are also consistent with the alternative possibility that the assessment of learned distractor suppression in the current paradigm originally designed for investigations at the group level, results in a learning measure that is unreliable at the individual level. We observed substantial inter-individual variance, but apart from low variance also measurement error can contribute to low reliability by introducing non-systematic changes in individuals’ scores between test sessions. Indeed, the fact that reliability within the same session was also found to be weak suggests that measurement error is an issue.

The finding that AC had in general higher reliability (both within and between sessions) than LDS can be partly explained by the fact that AC and LDS are not independent: the magnitude of AC (which was found to be only moderately reliable itself) co-determines the magnitude of LDS. For example, Failing and Theeuwes (2020) showed that salient distractors are suppressed more than less-salient distractors, at least when both their saliency signals were in the same (color) dimension (Liesefeld et al., 2019). Furthermore, van Moorselaar et al. (2021) showed correlations of about .44 between individual-level measures of AC and LDS obtained in different blocks. This strong relationship between the individual-level measures of AC and LDS could in fact completely explain away the test–retest correlation for distractor suppression. The mediation analysis we conducted showed that the relationship between Session 1 and Session 2 distractor suppression measures was no longer statistically significant after considering the averaged attentional capture measure of each participant. This suggests that the current way we measure statistical learning in the context of distractor suppression (as is typically done with difference scores) cannot be disentangled from the overall interference effect of an individual. Thus, any studies reporting a correlation between learned distractor suppression and other cognitive measures may suffer from this confound.

Whereas there is no golden standard for what is considered “good enough reliability”, measures in other facets of SL and standard cognitive tasks of working memory and attentional control show generally similar to substantially higher test–retest results as what we showed here (see Table 3). From a psychometric perspective, the observed estimates indicate that there is plenty of room for improvement and call for caution in using the existing statistical learning singleton paradigm with its commonly used measures for studying individual differences. Since the correlation that can be observed between two measures in upper-bounded by their reliability $({\rho }_{xy}= \sqrt{{\rho }_{x}* {\rho }_{y}}$), we cannot expect to observe high correlations with the LDS measure, even if the real correlation is high. Let us illustrate this with an example: imagine we want to correlate LDS with a measure of VSL which has a test–retest reliability of 0.68 (Siegelman et al., 2018), then the upper-bound of the correlation would be estimated ${\rho }_{xy}=\sqrt{0.26* 0.68 }=0.42$ (based on our full sample point estimate or test–retest reliability) at best and as low as ${\rho }_{xy}=\sqrt{0.02* 0.68 }=0.12$ (based on our point estimate of test–retest reliability after removing bivariate outliers, which is close to the lower bound of the confidence interval for the full sample). Whereas robust at the group level, AC but even more so measures of learned suppression are still limited as indices of individual-level performance with potential predictive validity and hence not optimally suited for testing theories regarding individual differences.

**Table 3:** Examples of test–retest reliability scores (*r* coefficients) of other cognitive tests

Visual Statistical Learning	Triplet learning (Siegelman & Frost, 2015; Siegelman et al., 2018)	0.58, 0.68
	Hebb repetition (Bogaerts et al., 2018)	~ 0–0.40
Working Memory	Lateralized Change Detection (Dai et al., 2019)	0.5–0.7
Attentional Control	Color Stroop (Hedge et al., 2018; Siegrist, 1995, 1997)	0.7
	Sustained Attention to Response Task (SART) (Robertson et al., 1997)	0.76
	Stop-Signal Task (SSRT) (Hedge et al., 2018)	0.4

Whereas not the main aim of the study, our dataset also allowed us to investigate correlations between different measures within each session. We found that target suppression measures did not significantly correlate with distractor suppression measures, which is at odds with the findings presented in Ferrante et al. (2018). They showed a correlation of 0.67 between the direct effect of SL on Distractor Filtering (what we call distractor suppression) and the indirect effect of SL on Target Selection (what we call target suppression), which they interpret as evidence that both processes share neuronal substrates. In the current study employing a larger sample, we found much weaker correlation coefficients that did not reach significance (*r* = 0.12, *p* = 0.289 in Session 1; and *r* = 0.22, *p* = 0.052 in Session 2). The problem with low reliability is a double-edged sword (Bogaerts et al., 2018) so that the discrepancy between results may suggest that a spurious correlation had been found while at the same time, the inclusion of a poorly reliable measure may be the culprit for not finding a correlation even if there actually is a true correlation between two measures. Indeed, given the results of our present study we may attribute this lack of consistency in the findings of different labs to unreliable measurement of the phenomena in question. In addition, we cannot exclude the possibility that this discrepancy between our results can be attributed to a difference between paradigms or lab vs. online settings.

### Future directions

In the current investigation, we have evaluated the psychometric properties of AC and LDS in the typical additional singleton task (Theeuwes, 1992) with a single high-probability distractor location as it has been used in multiple recent studies (e.g., Wang & Theeuwes, 2018a, b, 2020; Ferrante et al., 2018; van Moorselaar & Slagter, 2019, van Moorselaar et al., 2021; Di Caro & Della Libera, 2021; etc.). One limitation of our study was the large dropout between test and retest, resulting in somewhat lower precision of our estimates (see also Schönbrodt & Perugini, 2013). In addition, the current study focused specifically on spatial suppression of color singleton distractors and our findings hence do not preclude that a task with other parameters than the one under investigation here (e.g.: with different-modality distractors; a different probability distribution of the distractor locations; target regularities; an alternative task length) would perhaps fare better in terms of its psychometric properties.

One reason for the limited reliability we observed might be that AC and LDS measures are based on averages over the whole task, during which only one high-probability location was suppressed. Potentially more reliable indexing of suppression might be achieved by taking the average of several suppression “episodes” (i.e., when periodically changing the high-probability location during the task, thus requiring the suppression of new locations; e.g., Wang & Theeuwes, 2020). Using several suppression episodes may also allow for the computation of new individual metrics of LDS related to the speed of learning, such as the number of trials needed before a substantial difference in RTs can be observed between high-probability and low-probability locations. However, a possible disadvantage of such an approach might be the ‘overspill’ from previously suppressed locations. In addition, the ability to switch to new regularities may have substantial inter-individual variation on its own but may be quite a different measure of SL than the magnitude of suppression. Furthermore, having a longer study or averaging the data of several experimental sessions may provide more accurate within-subject estimates. Another common way to reduce unwanted noise for the calculation of reliability scores would be to present all participants with the exact same (fixed) trial sequence.

The reliability estimates we found could be used to inform better estimates of correlations between AC and other measures in future studies, by correcting for the imperfect reliability (e.g., Hedge et al., 2018). Moreover, from a practical point of view, our findings pave the way for the development of more reliable measures of LDS. As suggested above, future studies may try: using eye-tracking for better sensitivity; switching between and/or extinguishing high-probability locations to measure learning rate and flexibility; using very well-trained participants; aggregating scores from several sessions; using integrated measures well-suited for the experimental task, etc. Finding more reliable measures will allow further research into the factors underlying individual differences in LDS, which can ultimately lead to a better understanding of the variation in attentional capacities between individuals.

## Data Availability

The datasets and materials generated during the current study are available in the Open Science Foundation repository: https://osf.io/stvpf/?view_only=31c7881b3f534686ac3589504812f057

## Footnotes

1 In an additional control analysis, we calculated the test–retest coefficients including only the 66 participants for whom the high-probability location was different between test and retest. Test–retest reliability estimates were near-identical to what we observed for the full sample, hence we only report the latter.

## Code availability

Code is available in an Open Science Foundation repository: https://osf.io/stvpf/?view_only=31c7881b3f534686ac3589504812f057
